# Diagnostic criteria for temporomandibular disorders: self-instruction or formal training and calibration?

**DOI:** 10.1186/s10194-015-0505-9

**Published:** 2015-03-25

**Authors:** Larissa Soares Reis Vilanova, Renata Cunha Matheus Rodrigues Garcia, Thomas List, Per Alstergren

**Affiliations:** Department of Prosthodontics and Periodontology, University of Campinas, Piracicaba Dental School, Piracicaba, Brazil; Department of Orofacial Pain and Jaw Function, Faculty of Odontology, Malmö University, Malmö, Sweden; Scandinavian Center for Orofacial Neurosciences (SCON), Malmö, Sweden; Skåne University Hospital, Specialized Pain Rehabilitation, Lund, Sweden

**Keywords:** Diagnosis, Orofacial pain, Pain, Reliability, Temporomandibular disorders

## Abstract

**Background:**

To investigate the difference in diagnostic reliability between self-instructed examiners and examiners taught in a Diagnostic Criteria for Temporomandibular Disorders (DC/TMD) course and if the reliability of self-instructed examiners improves after the course.

**Methods:**

Six examiners were divided into three groups: (1) formal two-day training and calibration course at a DC/TMD training center (Course group), (2) self-teaching through documents and movie (Self group) with three examiners on each and the Self group later participated in the course (Self + course group). Each group examined sixteen subjects, total of 48 volunteers (36 patients with TMD and 12 asymptomatic) and the reliabilities in relation to the diagnoses derived by a Reference Standard Examiner were compared by Cohen’s Kappa coefficient.

**Results:**

The reliability was good to excellent in all three groups of examiners for all DC/TMD diagnoses, except for *Myofascial pain with referral* in the Self + course group. The course seemed to improve the reliability regarding *Myalgia* and *Arthralgia* at the same time as the examiners experienced the course to be valuable for self-perceived ability and confidence.

**Conclusions:**

This study shows that the diagnostic reliability of formal DC/TMD training and calibration and DC/TMD self-instruction are similar, except for subgroups of *Myalgia*. Thus, self-instruction seems to be possible to use to diagnose the most common TMDs in general dental practice. The course further improves the reliability regarding *Myalgia* and *Arthralgia* at the same time as the examiners experienced the course to be valuable for self-perceived ability and confidence.

## Background

Orofacial pain (OFP) and temporomandibular disorders (TMD) are conditions that affect more than 10% of the population [1,2]. Clinically, OFP/TMD is characterized by pain or dysfunction of the masticatory muscles, temporomandibular joint (TMJ) and/or related structures [3,4]. OFP/TMD is to various degrees covered in diagnostic classifications by for example American Association of Orofacial Pain (AAOP) [5], International Headache Society (ICHS) classification [6], International Association for the Study of Pain (IASP) [7], Classification of Chronic Pain and International Statistical Classification of Diseases and Related Health Problems (ICD-10) [8]. In 1992, the Research Diagnostic Criteria for Temporomandibular Disorders (RDC/TMD) was published. RDC/TMD was developed to cover the most common TMDs and incorporated two axes: Axis I covered the clinical condition and Axis II the psychosocial status and pain-related disability according to the biopsychosocial model of chronic pain [9]. The RDC/TMD has been used extensively over the last two decades. Reliability and validity have been widely studied [10-14] but the use of RDC/TMD has also been criticized.

Recently, a validated development of the RDC/TMD was published [15]. The Diagnostic Criteria for TMD (DC/TMD) provides a comprehensive assessment of the most common TMD conditions, based on the biopsychosocial model of chronic pain [15]. The DC/TMD similarly comprises two axes which the DC/TMD axis I protocol includes reliable, strictly specified and valid diagnostic criteria for the most common pain-related TMDs and intra-articular disorders [15]. Regarding Axis II, other studies have shown that the original RDC/TMD biobehavioral measures are incomplete in terms of prediction of disease course [2,5,15]. The DC/TMD instruments was therefore developed from RDC/TMD [15]. The DC/TMD is intended to be used in general dentistry as a validated tool to diagnose the most common OFP/TMD conditions.

In a research setting high operator reliability is important [13,16-18]. DC/TMD training and calibration can be conducted on three levels depending on the purpose of its use, e.g. general dentistry, speciality clinics or research. The training and calibration levels span from self-instruction via an instruction video and reading the documentation to a comprehensive two-day training and calibration course. This course should be given by a DC/TMD Training Center. A reliability assessment day can also be included. The latter level is of course very time- and resource-consuming and only allows three persons at a time to train, calibrate and assess their reliability at each occasion. In order to promote fast dissemination of the DC/TMD for clinical or research use, especially in general dentistry, self-instruction that gives acceptable diagnostic reliability compared to the training and calibration course would be ideal.

The strict examination and diagnostic procedures in DC/TMD require a certain amount of training. However, one main aim of the DC/TMD remains that it should be simple to learn and adopt, while still showing an acceptable reliability on a diagnosis level [15]. Training and calibration of examiners has previously, however, been shown to improve diagnostic reliability and accuracy [19-22].

The aim of this study was to investigate the difference in diagnostic reliability between self-instructed examiners and examiners taught by formal training and calibration in DC/TMD as well as if the reliability of self-instructed examiners improves after taking the course.

## Methods

### Setting

Reliability assessment data was gathered at three occasions at two OFP/TMD clinical centers in Linköping and Kalmar, Sweden. The first reliability assessment was performed after a two-day course in DC/TMD (Course group), the second was performed with self-instructed subjects (Self group) and the third occasion (three months after the second occasion) was performed after the self-instructed subjects also participated in the course (Self + Course group).

### Subjects

This study involved convenience samples of 36 patients referred for OFP/TMD examination and treatment to the two OFP/TMD clinical centers as well as 12 healthy individuals (Table 1). The inclusion criteria were participants presenting with TMD symptoms or healthy individuals without current or previous OFP/TMD symptoms. Exclusion criteria for both groups were individuals with age below 18 years and severe physical disease with ASA class ≥3 (cardiovascular, renal, pulmonary or autoimmune disease or malignancy), psychiatric disease (bipolar disorder, ADHD, autism spectrum disorders, anorexia nervosa, bulimia nervosa, schizophrenia and personality disorders whereas depression or anxiety disorders does not exclude the subject).

**Table 1:** **Demographic characteristics of patients with temporomandibular disorders and healthy individuals in DC/TMD reliability assessments**

		**Group**			
		**Course**	**Self**	**Self + course**	***P***
**Age**	*Years*	48 (42/68)	52 (30/58)	53 (47/61)	0.709
**Gender**	*Men*	7	2	4	0.135
	*Women*	9	14	12	
**Participants**	*Patients*	11	12	13	0.717
	*Healthy individuals*	5	4	3	
**Family situation**	*Single*	0	1	3	*0.040*
	*Married/Partner*	10	7	12	
	*Other (ex. Divorced/widow)*	6	8	1	
**Education**	*High school*	4	4	1	0.618
	*Gymnasium*	8	7	9	
	*College/University*	4	5	6	
**Occupation**	*Working/studying*	2	12	10	*0.005*
	*Retired/housewife/parental leave*	13	3	5	
	*Unemployed/ early retirement/sick listed*	1	1	1	
**Characteristic Pain Intensity**	*0-10*	2 (0/5)	4 (2/6)	4 (2/5)	0.646

DC/TMD = Diagnostic criteria for temporomandibular disorders. Age and characteristic pain intensity are reported as median (25th/75th percentile). P-vaules are from the Chi-square test or the Kruskall-Wallis ANOVA on ranks (age and characterstic pain intensity).

Before the examination all patients and healthy individuals answered the DC/TMD Axis II instruments used for assessment of psychosocial status and distress. The demographic characteristics of the sample regarding each group are described in Table 1. The subjects were informed about the project and signed a consent form. The project was approved by the regional Ethical Review Board in Lund, Sweden.

Sample sizes were established to demonstrate sufficient tolerance of repeated examination procedures for each patient and approved convenient clinical data to emerge as an important diagnostic method, as determined in published reports of RDC/TMD assessments [21,23,24]. The sample sizes used were based on previous studies [21,23,24] also to establish reliability of DC/TMD clinical examiners in distinguishing signs and symptoms of TMD. Furthermore, the reasonable time of practice in guiding patients was also analyzed. Patients and healthy individuals were evaluated by all examiners at each assessment.

### Examiners

**Figure 1:**
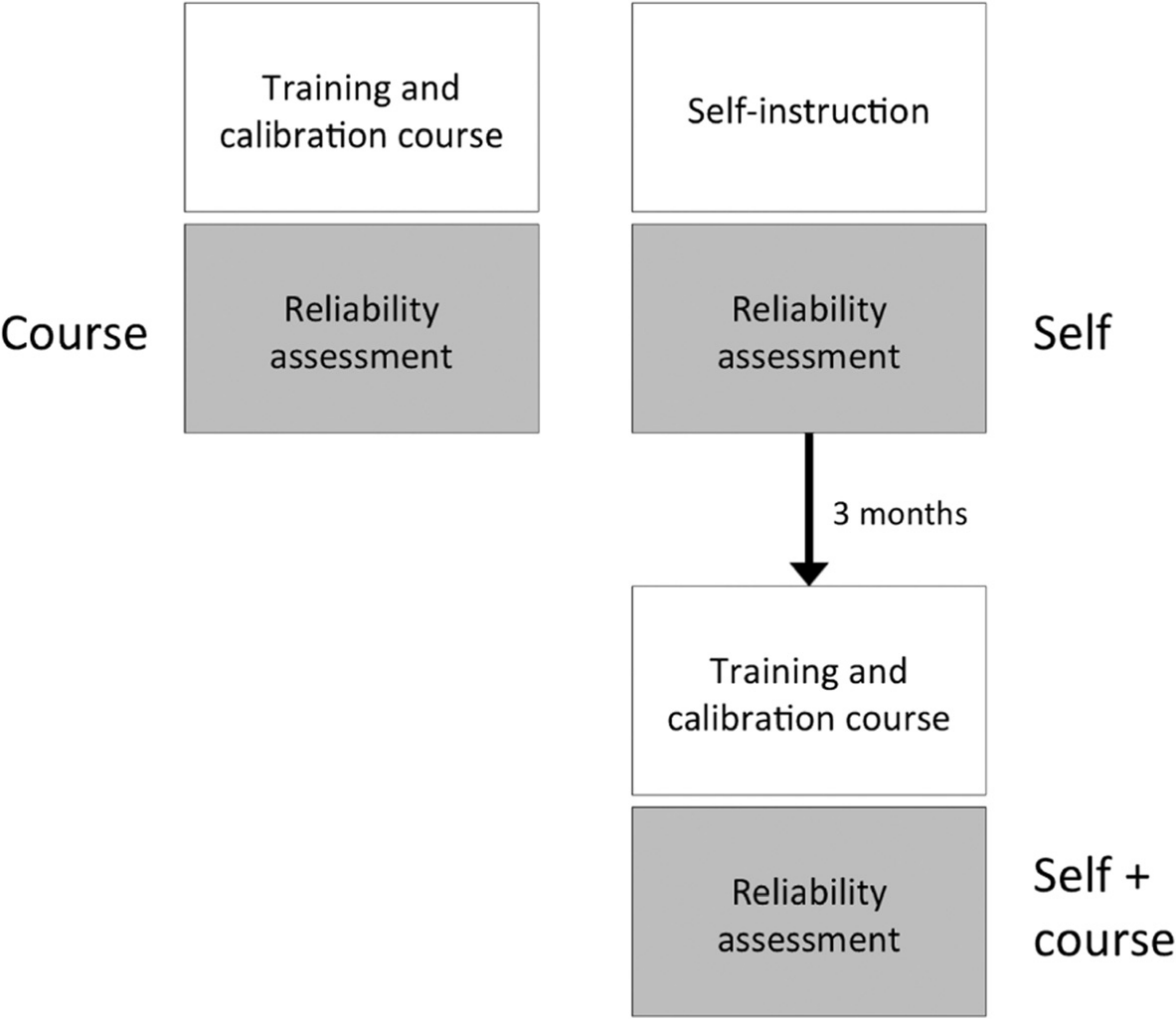
**Flow-chart of research methodology: Course, Self and Self + course groups in relation to reliability.**

Three OFP/TMD specialists and three general practitioners were included as examiners in a study design similar to a previous study [10] (Figure 1). The Course group consisted of three female specialists in OFP/TMD (mean age 54 years) and the Self/Self + course group comprised one male and two female general practitioners (mean age 31 years). Since DC/TMD is aimed to be used in the general dentistry, general practitioners were intentionally included. This allowed for testing of the performance of general practitioner, after self-instruction and after the course, compared to the performance of experienced OFP/TMD specialists after their course. An OFP/TMD specialist from the DC/TMD Training and Calibration Center in Malmö, Sweden acted as Reference Standard Examiner (RSE). In addition, another OFP/TMD specialist from the DC/TMD Training and Calibration Center in Malmö, Sweden participated as the Protocol Supervisor (PS).

### DC/TMD training and calibration course

Information and full documentation were sent to the participants prior to the course in order to ensure that they had the possibility to learn about DC/TMD and especially to learn and memorize all mandatory commands. The standardized two-day course in DC/TMD training and calibration comprised the following steps: the first half-day consisted of theoretical education about DC/TMD purpose, history and future as well as clinical diagnoses (Axis I) and psychosocial status and distress (Axis II). The second half-day included a detailed explanation of the clinical examination using the instruction movie and clinical training for the participants by performing the clinical examination on the RSE while the RSE gave immediate and detailed feedback. The morning of the second day started with clinical training of the participants by clinical examination of each other while the RSE was standing beside and giving immediate feedback. After that, clinical calibration of the participants was performed by clinical examination of patients where the RSE was present in the room and giving detailed feedback after completion of the procedure. The last afternoon included diagnostic exercises on patient cases, patient case discussions and discussions about how to implement DC/TMD in the clinical practice.

### DC/TMD self-instruction

The three operators in the Self Group were instructed to download the instruction movie as well as the full documentation and learn the DC/TMD examination procedure by themselves.

### Reliability assessment

The reliability on a diagnostic level of DC/TMD Axis I diagnoses, based on the questionnaire and clinical examination, was assessed during one day. The examiners individually examined all patients and healthy individuals, blinded from each other’s findings. An incomplete Latin square design was used such that order of the examiners was randomized to balance and minimize examiner’s examination order effects. The reliability assessment was performed in four two-hour blocks of DC/TMD examinations. Each examination was performed over a maximum of twenty minutes in order to allow the subject to rest at least ten minutes between examinations. The examiners moved between the participating patients and healthy individuals within each dental clinic setting, with the patient/healthy individual remaining seated in the same clinical operatory. A recorder was assigned to each operatory. Manuals were provided to the examiners which defined all variables and how they are assessed. All instructions to patients and healthy individuals were delivered by the examiner in Swedish, using standardized translations produced by DC/TMD Training and Calibration Center in Malmö, Sweden in accordance to accepted standards for producing such translations as required and adopted by the International DC/TMD Consortium [25].

### Protocol supervisor observation and feedback

The PS observed one examiner at a time during the clinical examinations and covered during the day all examiners, including the RSE. After each clinical examination, the PS gave detailed feedback to the examiner that was observed by the PS. After each two-hour block, the PS recorded feedback from the subjects and recorders regarding any differences between the examiners related to instructions, commands and procedures. This was summarized and forwarded to the examiners after each two-hour block.

### Self-perceived ability to perform DC/TMD diagnostics and satisfaction with DC/TMD learning

After the reliability assessment, the examiners were asked to rate their degree of agreement with statements about their self-perceived ability in DC/TMD diagnosis (regarding several aspects of the DC/TMD procedure) and their satisfaction regarding how they learnt various aspects of the DC/TMD procedure. The questionnaire comprised 10 statements with end-points “No ability” and “Very high ability” as well as “Not satified” to “very satisfied”.

The Self + course group completed a separate questionnaire after their second reliability assessment. The examiners were asked to rate to what extent the course improved (end-points “Not at all” and “To a very high degree”) their ability and learning regarding various aspects of the DC/TMD procedure.

### DC/TMD diagnoses

The DC/TMD clinical data, as described above, were used to derive DC/TMD diagnoses by the use of the DC/TMD diagnostic algorithms [15]. The following DC/TMD Axis I diagnoses were derived:

#### Pain-related TMD and headache

- Local myalgia (per subject)
- Myofascial pain with referral (per subject)
- Myalgia (local myalgia and myofascial pain with referral; per subject)
- Arthralgia (per joint)
- Headache attributed to TMD (per subject)

#### Intra-articular joint disorders and Degenerative joint disorder

- Disc displacement with reduction (per joint)
- Disc displacement with reduction with intermittent locking (per joint)
- Disc displacement without reduction (per joint)
- Disc displacement without reduction without limited opening (per joint)
- Degenerative joint disease (per joint)

### Data analysis

The diagnoses derived from the clinical data from the examiners, i.e. based on responses to the questionnaires as well as data from the clinical examination, were compared to those derived from the RSE clinical data.

The Cohen's kappa coefficient was used to calculate the reliability of the DC/TMD diagnoses. TMJ specific diagnoses were treated as independent observations, *i.e.* two diagnoses per individual: one for the left and/or one for the right TMJ, for each of the possible diagnoses. The reliability of DC/TMD diagnoses was determined for each reliability assessment. Kappa values as follows were interpreted as: < 0: less than chance agreement, 0.01 - 0.20: slight agreement, 0.21 - 0.40: fair agreement, 0.41 - 0.60: moderate agreement, 0.61 - 0.80: substantial agreement and 0.81 - –0.99: almost perfect agreement [26,27]. Statistics were performed using Stata software, version 12-SE (Stata Corp., College Station, TX, USA).

The Chi-square test was used to calculate the significance for differences in distribution of gender, participants, family situation, education level and occupation between the sites. The Kruskall-Wallis ANOVA on ranks was used to calculate the significance of a difference in age and characteristic pain intensity between the patients and healthy individuals participating at the reliability assessment in each group. A probability level of *P* < 0.05 was considered as significant.

## Results

### DC/TMD diagnoses

Table 2 shows the distribution of diagnoses derived at each reliability assessment by the RSE. There was a significantly higher prevalence of the diagnosis *Arthralgia* in the patients and healthy individuals at the reliability assessment for the Self + course group compared to those included at the Self group and Course group reliability assessments (*P* = 0.027). No other significant difference regarding distribution of diagnoses was found.

**Table 2:** **Prevalence of DC/TMD diagnoses as derived from data gathered by the reference standard examiner**

	**Group**			
	**Course**	**Self**	**Self + course**	
**Diagnosis**	**n (%)**	**n (%)**	**n (%)**	***P***
**Pain-related TMD and headache**				
Myalgia	7 (43%)	8 (50%)	13 (81%)	0.070
Local myalgia	4 (25%)	0	4 (25%)	0.091
Myofascial pain with referral	3 (18%)	8 (50%)	9 (56%)	0.070
Arthralgia	9 (28%)	9 (28%)	18 (56%)	0.027
Headache attributed to TMD	5 (31%)	4 (25%)	6 (37%)	0.789
**Intra-articular joint disorders**				
Degenerative joint disease	9 (28%)	7 (21%)	7 (21%)	0.796
Disc displacement with reduction	6 (18%)	3 (9%)	8 (25%)	0.257
Disc displacement with reduction, with intermittent locking	0	0	2 (6%)	0.130
Disc displacement without reduction	0	2 (6%)	0	0.364
Disc displacement without reduction, with limited opening	0	2 (6%)	0	0.132
**No DC/TMD diagnosis**	5 (31%)	4 (25%)	3 (18%)	0.751

DC/TMD = Diagnostic criteria for temporomandibular disorders; n = number of observations.

The diagnosis Myalgia is the diagnoses Local myalgia and Myofascial pain with referral combined to be used in general practice.

### Diagnostic reliability

The Kappa values (median and range) for the intra-operator reliability compared to the RSE for each investigated DC/TMD diagnosis are shown in Table 3. The reliability was moderate or better for all diagnoses except for *Local myalgia* and *Myofascial pain with referral* for the Self + course group. Disc-related diagnoses other than *Disc displacement with reduction* were not possible to compare between the examiner groups due to the low prevalence in the participating subjects preventing the calculation of Kappa values.

**Table 3:** **Reliability (Cohen’s Kappa values) of DC/TMD diagnoses compared to the reference standard examiner**

	**Course**		**Self**		**Self + course**	
**Diagnosis**	**Median**	**Range**	**Median**	**Range**	**Median**	**Range**
**Pain-related TMD and headache**						
Myalgia	0.91	(0.88 - 1.00)	0.65	(0.50 - 0.75)	1.00	(0.82 - 1.00)
Local myalgia	0.84	(0.71 - 1.00)	n.a.	n.a.	0.43	(0.14 - 0.70)
Myofascial pain with referral	0.76	(0.60 - 0.81)	0.62	(0.35 - 0.70)	0.26	(0.07 - 0.70)
Arthralgia	0.66	(0.42 - 0.87)	0.47	(0.47 - 0.60)	0.74	(0.62 - 0.81)
Headache attributed to TMD	0.84	(0.70 - 0.86)	0.82	(0.60 - 1.00)	1.00	(0.87 - 1.00)
**Intra-articular joint disorders**						
Degenerative joint disease	0.66	(0.66 - 0.92)	0.81	(0.71 - 0.81)	0.73	(0.73 - 0.81)
Disc displacement with reduction	0.44	(0.24 - 0.61)	0.52	(0.35 - 0.84)	0.73	(0.73 - 0.81)

DC/TMD = Diagnostic criteria for temporomandibular disorders. n.a. = not applicable (due to prevalence = 0). The diagnosis *Myalgia* is the diagnoses *Local myalgia* and *Myofascial pain with referral* combined to be used in general practice.

The low reliability for *Local myalgia* and *Myofascial pain with referral* in the Self + Course group motivated a deeper analysis. The number of patients diagnosed with *Myofascial pain with referral* by the RSE was higher than patients diagnosed by the examiners. Accordingly, the number of patients diagnosed with *Local myalgia* by the RSE was lower than patients diagnosed by the examiners. This indicates a discrepancy between the RSE and examiners regarding assessment of referred pain. An analysis of the number of sites with referred pain showed that there was a relation between the findings of the RSE and the examiners but that the RSE in general found more sites with referred pain (data not shown).

The lower Kappa values for all three groups regarding *Arthralgia* also motivated a separate analysis. The degree of agreement between the RSE and the examiners for separate variables related to the DC/TMD diagnoses *Arthralgia* is presented in Table 4. The Self group showed a lower agreement than the other groups regarding pain location, TMJ pain on movement and TMJ palpation pain than the other groups.

**Table 4:** **Agreement between three examiners in each group regarding findings related to the DC/TMD diagnosis Arthralgia**

			**Agreement with RSE**		
			**C**	**S**	**S + C**
ARTHRALGIA					
**TMJ pain location**	*Agreement*	*%*	92	76	91
	*Disagreement*	*%*	8	24	9
**TMJ pain on movement**	*Agreement*	*%*	76	67	88
	*Disagreement*	*%*	24	33	12
**TMJ palpation pain**	*Agreement*	*%*	91	70	96
	*Disagreement*	*%*	9	31	4

DC/TMD = Diagnostic criteria for temporomandibular disorders.

C = Course Group, S = Self Group, S + C = Self + Course Group.

### Protocol supervisor observations

The subjects reported that the examiners initially used individual words but there was an early and substantial improvement in all three groups to match the RSE. The subject’s understanding of the commands and what the subjects expected to do were clear. For procedures, there were minor initial differences between the examiners in the Course group and Self + course group. In the Self group, there were initial differences regarding force used as well as sites and time for palpation, differences that disappeared early during the day after individual feedback from the PS. For the Self group, the PS noticed that approximately 50% more feedback was required and that the variation between the examiners were larger in the beginning of the day. The examiners were very similar to the RSE at the last two-hour block of the day regarding commands and instructions.

The recorders noticed that the communication was adequate in general for the Course group and the Self + course group. However, the communication was unsatisfactory and inadequate from the examiners in the Self group for the first subjects examined. The recorders noticed a substantial improvement in communication between the reliability assessments for the Self group and the Self + course group.

### Self-evaluation of diagnostic ability and learning satisfaction

The self-evaluation by the examiners in the Course group and Self group regarding confidence in their ability to perform the clinical examination, derive DC/TMD Axis I diagnoses and to interpret the Axis II instruments as well as their satisfaction about their learning of the DC/TMD procedure is presented in Table 5. The examiners evaluated their ability and satisfaction to be high and very similar, in general.

**Table 5:** **Median and range of self-evaluation scores**

	**Ability**			**Satisfaction**		
	**Course**	**Self**	**Self + course**	**Course**	**Self**	**Self + course**
**Clinical examination**						
Give correct instructions to the patient	8 (7–8)	9 (8–10)	9 (8–10)	7 (6–8)	9 (7–10)	8 (8–10)
Identify pain localization	9 (8–9)	8 (6–10)	7 (5–8)	8 (8–9)	9 (6–10)	8 (8–10)
Measurements (range of motion, overbite, etc.)	9 (9–10)	9 (8–10)	9 (8–10)	9 (9–10)	9 (6–10)	8 (8–10)
Assessment of temporomandibular joint sounds	7 (7–8)	7 (5–8)	8 (7–10)	8 (7–9)	7 (5–8)	8 (8–10)
Palpation of muscles	9 (8–9)	8 (6–8)	7 (5–9)	9 (8–9)	7 (3–9)	7 (8–10)
Palpation of temporomandibular joints	9 (8–9)	8 (6–8)	7 (5–9)	8 (8–9)	7 (3–9)	7 (8–10)
Identifying familiar pain	9 (8–9)	8 (6–10)	8 (8–10)	9 (9–10)	9 (5–9)	8 (8–10)
Identifying referred pain	8 (8–9)	8 (6–10)	7 (5–8)	9 (8–10)	9 (3–10)	7 (8–10)
**Diagnostics, etc.**						
Derive DC/TMD-diagnosis (Axis I)	7 (6–8)	7 (6–8)	8 (7–8)	7 (6–8)	8 (6–10)	8 (7–10)
Interpretation of instruments used to assess psychosocial factors (Axis II)	8 (7–9)	7 (5–8)	9 (6–10)	8 (7–9)	7 (5–8)	8 (5–10)

0 = not at all, 10 = to a very large degree.

The Self + course group scored that the course improved their confidence in their ability to perform the clinical examination, derive DC/TMD Axis I diagnoses and to interpret the Axis II instruments to a great extent in general.

## Discussion

The results of the present study show that diagnostic reliability was high after self-teaching DC/TMD or participating in the two-day DC/TMD training and calibration course. The Self group tended to improved their reliability regarding *Myalgia* and *Arthralgia* after participating in the two-day DC/TMD training and calibration course. For potential use in general practice, the diagnosis *Myalgia* showed substantial to almost perfect reliability in all groups.

The validated DC/TMD with established sensitivity and specificity for the most common OFP/TMD conditions comprise strict clinical procedures and diagnostic criteria. These procedures must be memorized and trained in order to ensure reliability and to achieve the established sensitivity and specificity. The formalized two-day training and calibration course is likely an optimal way to learn DC/TMD but it is time- and resource-consuming. It also requires participation of a RSE or PS from a DC/TMD Training and Calibration Center. This severely limits the availability of such courses, at least today. To promote spread of the use of DC/TMD, not the least among general practitioners that wants to use this in their clinical practice, a less resource-consuming self-instruction that achieves acceptable diagnostic reliability would be highly advantageous.

The self-instruction, as applied in this study, means that the participants download an instruction movie as well as the DC/TMD documentation. The results in this study are thus partly based on the pedagogical quality of this material. This study gives indications on how the self-instruction material can be improved. When accomplished, such improvement should have the possibility to give even higher reliability.

### Diagnostic reliability

The diagnostic reliability was moderate to almost perfect after self-teaching DC/TMD or participating in the two-day DC/TMD training and calibration course, indicating that self-teaching is sufficient in order to achieve adequate reliability for the investigated diagnoses but that the reliability seems to be improved further by the course.

The differences in reliability between the groups regarding *Local myalgia* and *Myofascial pain with referral* is likely explained by differences in assessing referred pain, according to the deeper analysis. As a consequence, assessment of referred pain should be improved and carefully taught in DC/TMD training. We cannot explain why the Self + course group showed the lowest reliability for *Local myalgia* and *Myofascial pain with referral*. The reliability for *Myalgia* was, however, very high showing that the examiners were very good at identifying patients with masticatory muscle pain in general but not as good to divide the patients into subgroups of muscle pain with or without referred pain. This was surprising since the examiners that participated in the Self and Self + course groups had learnt DC/TMD by themselves as well as taken the two-day course.

This is the first study regarding diagnostic reliability of the recently published DC/TMD criteria.^15^ Examiner training and calibration, rather than professional experience, is the most important factor for reliable diagnosis of TMD symptoms using RDC/TMD.^20^ Re-training and re-calibration of examiners in RDC/TMD diagnostics improves reliability of most of clinical variables [16,19,28] whereas experienced clinicians that did not participate in training and calibration showed low reliability [9]. The Self group improved their reliability regarding *Myalgia* and *Arthralgia* after participating in the two-day DC/TMD training and calibration course. This points to a need for the course in situations where the highest possible reliability is important, like multi-center research projects.

The reliability for *Arthralgia* was substantial for the Course group and the Self + course group and only moderate for the Self group. The difference in reliability is probably not due to a too low prevalence in one or more groups. Our raw data indicate that the disagreement between the RSE and the examiners in the Self group was due to differences in recording of TMJ pain during the last 30 days, TMJ pain on movements and TMJ palpation pain. The Course group and the Self + course group showed the highest agreement to the RSE, indicating a significant contribution of the training and calibration course to the correct DC/TMD assessment of TMJ pain. This part must thus be thoroughly emphasized in the final self-teaching material.

Diagnosis of *Disc displacement with reduction* showed a substantial reliability for the Self + course group but a moderate reliability for both the Course group and the Self group. The Kappa value is affected by the prevalence of the finding under consideration and for rare findings, low values of kappa may not necessarily reflect low rates of overall agreement [29]. Among the subject included for the Course group and the Self group, the prevalence of *Disc displacement with reduction* was fairly low, 9 and 18% respectively. In the subjects in the Self + course group the prevalence was higher, 25%. The difference in prevalence may therefore be one reason for the difference in reliability between these groups. The deeper analysis of which clinical variable that may have contributed to this difference did not show any particular differences between the groups, supporting the fact that it was the prevalence that explains the difference in reliability.

Taken together, self-teaching DC/TMD seems to result in sufficient actual and self-perceived ability on a diagnostic level to derive the diagnoses *Myalgia*, *Arthralgia*, *Degenerative joint disease*, *Disc displacement with reduction* and *Headache attributed to TMD*. This is important for promoting the dissemination of the use of DC/TMD, especially in general dentistry. If use of the subgroups *Local myalgia* and *Myofascial pain with referral* is important, for example in specialist clinics or certain research projects, the course is probably crucial to improve the reliability of these subdiagnoses as well as *Arthralgia*.

### Observations by the subjects, recorders and the protocol supervisor

The observations by the subjects, recorders and the PS indicate that the training and calibration course is important in order to assure that identical commands as well as palpation sites and forces are used, that the pain location is checked and updated throughout the examination and that the communication with the recorder is adequate from the first clinical examination. However, during the reliability assessment day the Self group improved these aspects and at the last examination session the examiners in the Self group were very similar to the RSE, just as the Course group and the Self + course group.

### Self-evaluation of the ability and learning of DC/TMD

The self-perceived ability in performing the DC/TMD procedure as well as the satisfaction of learning DC/TMD was in general high and with no apparent difference between the Self group and Course group. However, the Self + course group appraised that the course improved both their ability and satisfaction to a great extent. This is most probably due to the intense theoretical teaching and clinical training, including feedback and discussion, provided in the course. This means that the course is appreciated and has effects on the self-perceived ability in DC/TMD but that self-teaching is still sufficient for performing the DC/TMD procedure and deriving diagnoses from the clinical data.

### Improving the self-teaching material

Based on the findings of the present study, the instruction movie and documentation to be used for self-instruction should be improved to emphasize i) the need to update the pain location in the clinical form as the clinical examination proceeds, ii) the assessment of referred pain on palpation and iii) the assessment of pain location in the TMJ and masseter muscle or both.

## Conclusion

In conclusion, this study shows that the diagnostic reliability of formal DC/TMD training and calibration and DC/TMD self-instruction are similar, except for subgroups of *Myalgia*. Thus, self-instruction seems to be possible to use to diagnose the most common TMDs in dental practice. The course further improves the reliability regarding *Myalgia* and *Arthralgia* at the same time as the examiners experienced the course to be valuable for self-perceived ability and confidence.
